# A new species of *Labidocera* (Copepoda, Calanoida, Pontellidae) collected from Okinawa, southwestern Japan, with establishment of five Indo-West Pacific species groups in the *L. detruncata* species complex

**DOI:** 10.3897/zookeys.447.8171

**Published:** 2014-10-16

**Authors:** Takeshi Hirabayashi, Susumu Ohtsuka

**Affiliations:** 1 Takehara Marine Science Station, Setocuhi Field Center, Graduate School of Biosphere Science, Hiroshima University, 5-8-1 Minato-machi, Takehara, Hiroshima 725-0024, Japan

**Keywords:** Indo-West Pacific, *Labidocera*, Okinawa, Pontellidae, species group, taxonomy

## Abstract

*Labidocera churaumi* **sp. n.** is described from Okinawa, southwestern Japan. The female of the new species differs from other congeners in genital compound somite with right postero-lateral and left antero-lateral processes. The male is distinguished from other congeners by the structure of the fifth leg. This new species is assigned to a newly proposed species group, the *Labidocera madurae* species group, within the *Labidocera detruncata* species complex. In this species complex five Indo-West Pacific species groups are recognized (*cervi*, *detruncata*, *gangetica*, *madurae*, and *pavo*) and defined on the basis of difference in sexual dimorphism.

## Introduction

We have been carrying out intensive faunistic surveys of marine invertebrates around the Nansei Islands, in the subtropical region of southwestern Japan, since 1988 and have discovered many new crustacean taxa, especially copepods. For example the copepod order Platycopioida was first reported from the Indo-Pacific in this region in 1994 (Ohtsuka and Boxshall 1994). The systematics of shallow- and deep-water copepods from these waters, have been a major focus of research and some of discoveries have been important in elucidating aspects of the evolutionary history and zoogeography of copepods (Barr and Ohtsuka 1989, Ohtsuka et al. 1991, 1996, 1998, 2002, 2003, 2004, 2005, Barthélémy et al. 1998).

In May 2011 we found an undescribed species of the calanoid genus *Labidocera* Lubbock, 1853 (Family Pontellidae) from Okinawa Island and neighboring islands. It clearly belongs to the *Labidocera detruncata* species complex (Fleminger 1967, Greenwood and Othman 1979, Mulyadi 2002), due to its characteristic sexual dimorphism. This species complex mainly consists of coastal species from tropical and subtropical Indo-West Pacific waters and also two Atlantic species (Fleminger 1967, Greenwood and Othman 1979, Mulyadi 2002). The present paper provides a description of the new species, and remarks on species groups within the *detruncata* species complex.

## Materials and methods

A fish collection light (KU-5MB, MW50S-G, KOTO electric Co., Ltd.) was deployed at Naha Port (May 2011) and Tokashiki Port (May 2011) after sunset. Conical plankton nets (diameter 30 cm, mesh size 0.1 mm) were towed around the light several times. Copepod specimens were fixed with 10% neutralized formalin/seawater or 70% ethanol immediately after collection. Copepods were dissected under a binocular microscope and examined and illustrated using a compound microscope fitted with differential interference contrast lighting (Optiphoto, Nikon Co., Ltd.) and a drawing tube.

In describing the features of the new species, we have followed the terminology of Huys and Boxshall (1991). We followed Fleminger (1967) and Fleminger et al. (1982) about species complexes (=superspecies) and groups in the genus *Labidocera*.

Type specimens are deposited at the Kitakyushu Natural History and Human History Museum (KMNH IvR 500,734 – KMNH IvR 500,783).

## Systematics

### Order Calanoida Sars, 1903 Family Pontellidae Dana, 1853 Genus *Labidocera* Lubbock, 1853

#### Labidocera churaumi sp. n.

Taxon classificationAnimaliaCalanoidaPontellidae

http://zoobank.org/E4C58DC0-DDE3-4808-B7D1-5925D444280B

Figs 1 , 2 , 3 , 4

##### Material examined.

Tokashiki Port, Tokashiki Island, Okinawa Prefecture, Japan, (26°12'0.98"N; 127°22'8.77"E), 21 May 2011 (8 ♀♀, 3 ♂♂); (26°12'1.21"N; 127°22'10.20"E), 27 May 2012 (21♀♀, 11♂♂). Naha New Port, Okinawa Prefecture, Japan, (26°14'8.22"N; 127°40'47.56"E), 20 May 2011, (1 ♀, 6 ♂♂).

##### Types.

Holotype: 1♀, Tokashiki Port, 27 May 2012, whole specimen (KMNH IvR 500,759). Allotype: 1♂ Tokashiki Port, 27 May 2012, whole specimen (KMNH IvR 500,783). Paratypes: 1♀, 6♂♂, Naha New Port, 20 May 2011, whole specimen (♀ KMNH IvR 500,734; ♂♂ KMNH IvR 500,764-KMNH IvR 500,769); 8 ♀♀, 3 ♂♂, Tokashiki Port, 21 May 2011 partly dissected and mounted on 11 glass slides (♀♀KMNH IvR 500,735-KMNH IvR 500,742; ♂♂KMNH IvR 500,770-KMNH IvR 500,772); 20 ♀♀, 10♂♂, Tokashiki Port, 27 May 2012, whole specimen (♀♀KMNH IvR 500,743-KMNH IvR 500,758 and KMNH IvR 500,760-KMNH IvR 500,763; ♂♂KMNH IvR 500,773-KMNH IvR 500,782).

##### Type locality.

Tokashiki Port, Tokashiki Island, Okinawa Prefecture, Japan (26°12'1.21"N; 127°22'10.20"E).

##### Female.

**Figure 1.**
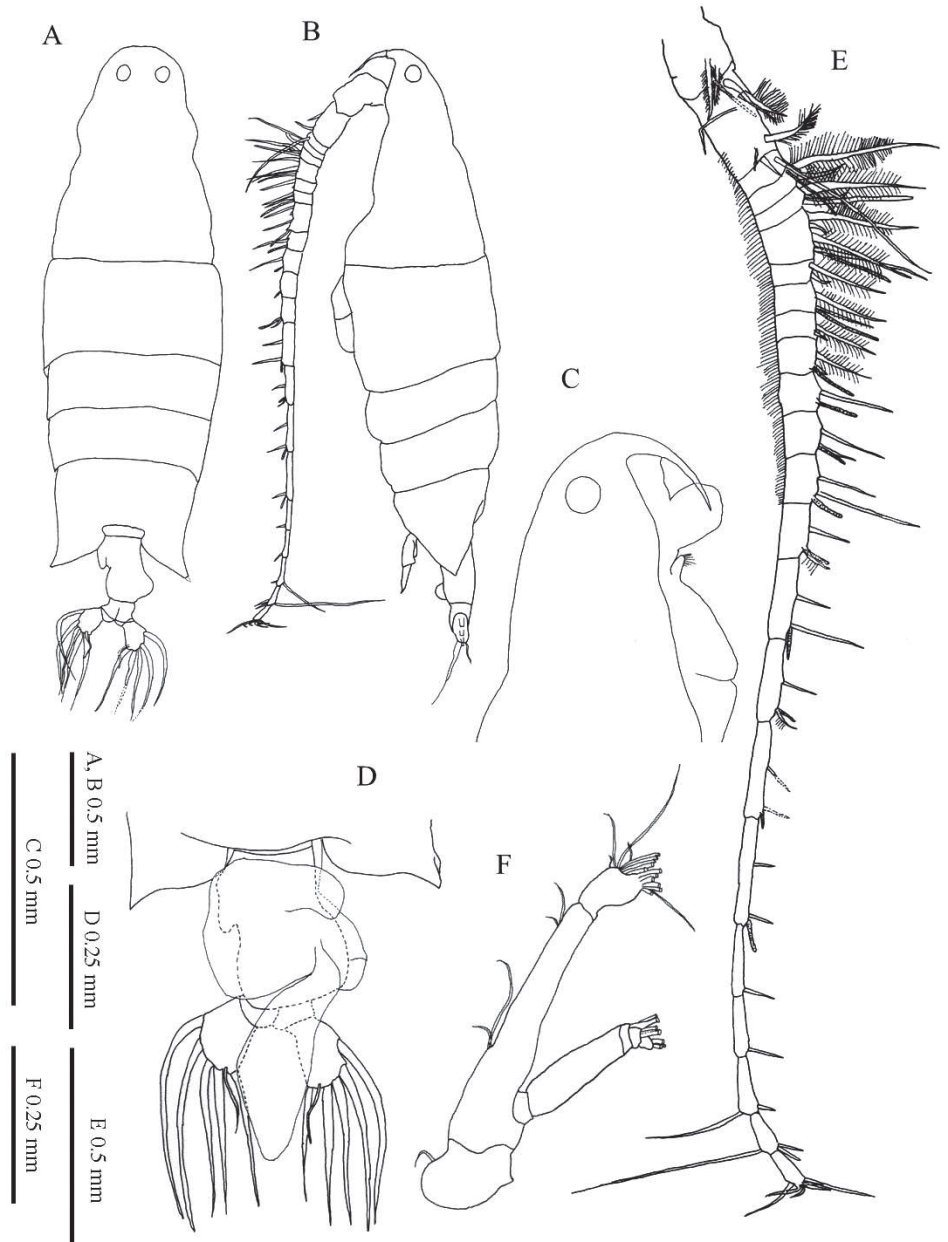
*Labidocera churaumi* sp. n., female (Paratype). **A** habitus, dorsal view **B** habitus, lateral view **C** anterior part of cephalosome, lateral view **D** urosome with outline of attached spermatophore and coupler, dorsal view **E** left antennule **F** antenna.

Body (Fig. 1A, B) length of females ranging between: 2225 and 2790 µm (average 2475 µm, n=29), measured from frontal margin of cephalosome to end of caudal rami excluding caudal setae. Ratio of prosome to urosome lengths 4:1, prosome length to width ratio 2.85:1. Cephalic profile rounded in dorsal view, without lateral cephalic hooks. Paired dorsal eyes with cuticular lenses; protuberant ventral eye extending anteroventrally between rostral processes (Fig. 1C). Rostrum bifid, directed posteroventrally. Posterior margins of prosome almost symmetrical in dorsal view, tapering to simple abbreviated, pointed process at each lateral corner. Urosome 2-segmented of highly characteristic shape. Genital compound somite strongly asymmetrical; anterior left surface with posteriorly-directed rod-like process and posterior right smoothly rounded. Spermatophore (Fig. 1D) attached dorsally to genital compound somite.

**Figure 2.**
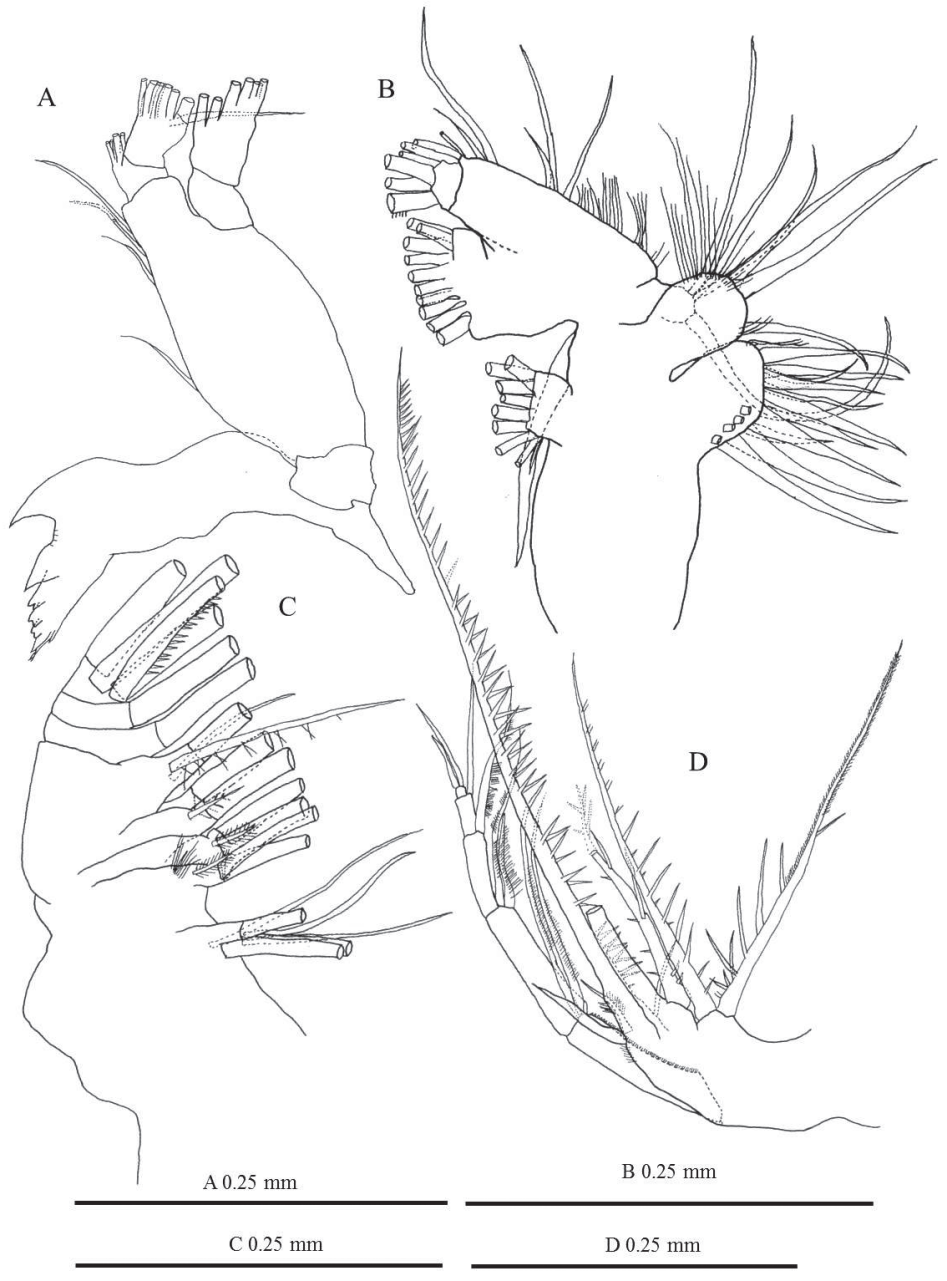
*Labidocera churaumi* sp. n., female (Paratype). **A** mandible **B** maxillule **C** maxilla **D** maxilliped.

Antennules (Fig. 1E) symmetrical, 23-segmented: segments armed as follows (Arabic numbers=setae; sp=spines, aes=aesthetasc): (I) 2+aes, (II-IV) 4+aes, (V) 2+aes, (VI) 2, (VII) 2+aes, (VIII-IX) 4+aes, (X) 2, (XI) 2+aes, (XII) 1+sp, (XIII) 1+sp+aes, (XIV) 1+sp+aes, (XV) 2+aes, (XVI) 2+aes, (XVII) 1+sp+aes, (XVIII) 2+aes, (XIX) 1+sp+aes, (XX) 2+aes, (XXI) 2+aes, (XXII) 1, (XXIII) 1, (XXIV) 1+sp, (XXV) 1+sp+aes, (XXVI-XXVIII) 6. Larger and longer setae on segments 3-6. Antenna (Fig. 1F) biramous: coxa with short plumose distal seta, basis and first endopodal segment fused to from elongate allobasis, setation formula 2, 2. Compound distal endopodal segment with 9 and 7 setae on proximal and distal lobes, respectively; exopod 5-segmented, setation formula 0, 0, 2, 2, 3. Mandible (Fig. 2A) with wide, heavily chitinized gnathobase; mandibular palp biramous, basis robust, armed with 4 inner setae. Endopod 2-segmented, first segment armed with 1 short and 3 long setae; second segment with 7 terminal setae. Exopod 2-segmented, first segment unarmed, second segment with 6 terminal setae. Mandibular gnathobase distal edge bearing 8 teeth comprising: from ventral margin 1 apical, 1 subapical, 3 compound medial, and 3 basal (see Fig. 2A); medial teeth with bifurcated cusps; dorsal end of gnathobase with 1 seta. Maxillule (Fig. 2B) praecoxal arthrite with 15 setal elements, 4 on posterior surface; coxal endite with 2 long and 1 short elements on endite and 9 setae on epipodite; basis with 3 setae on proximal and distal endites; and 1 large seta on basal exite; proximal endopod segment and endpod segment 2 incorporated into basis, proximal endopod segments with 2 setae, endopod segment 2 with 2 setae and distal endopod segment with 5 apical setae; exopod with 10 setae. Maxilla (Fig. 2C) with first praecoxal endite bearing 6 setae, second with 3 seta; coxa with 3 setae each on proximal and distal endites. Basis with 3 setae; endopod 3-segmented, setal formula of endopod: 1, 1, 4. Maxilliped (Fig. 2D) with praecoxa and coxa fused, three syncoxal endites well developed, with setal formula 2, 2, 4; endite setae strong, spinulose. Basis fringed with medial row of spiniform processes and 2 distal setae. Endopod 4-segmented, setal formula of endopod as: 2, 1, 1, 2.

**Figure 3.**
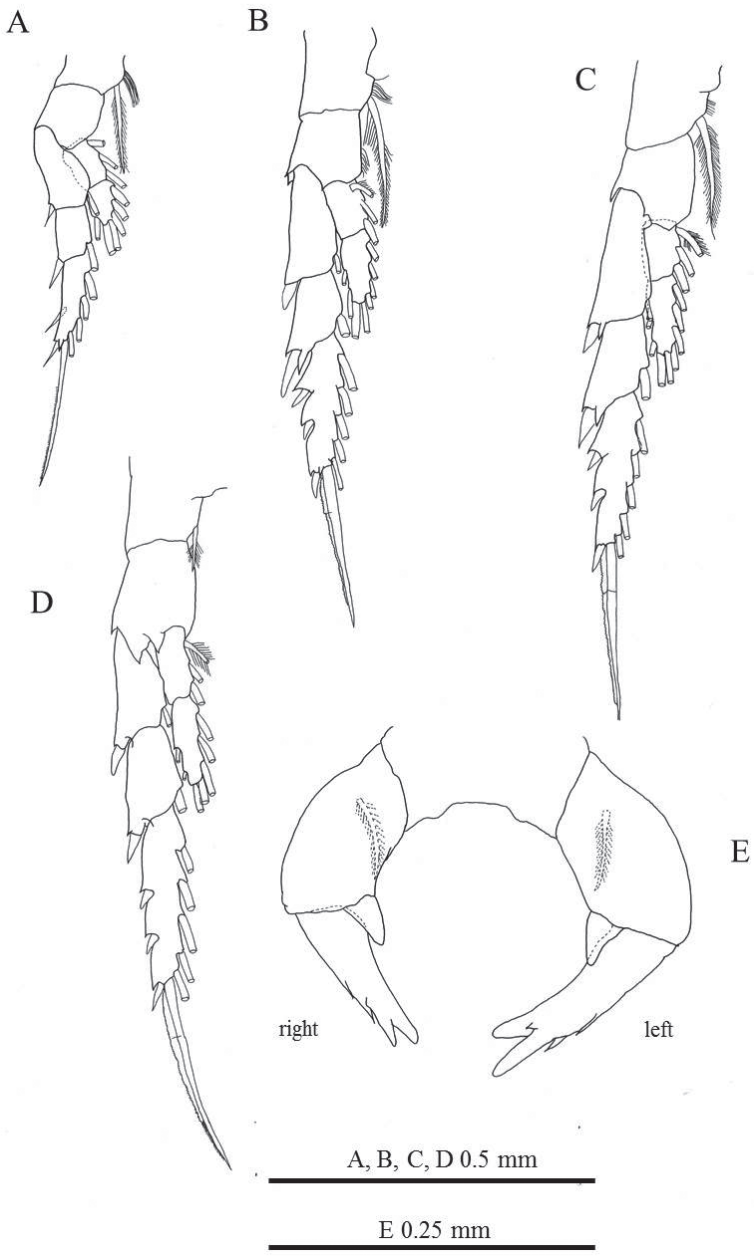
*Labidocera churaumi* sp. n., female (Paratype). **A** leg 1 **B** leg 2 **C** leg 3 **D** leg4 **E** leg 5.

Legs 1–4 (Fig. 3A–D) with 2-segmented endopods and 3-segmented exopods. Coxae with plumose inner seta. Seta and spine formula (Arabic numbers=setae, Roman numerals=spines) of legs 1–4 as follows:

**Seta and spine formula:** Arabic numbers=setae, Roman numerals=spines

	Coxa	Basis	Exopod	Endopod
Leg 1	0-1	0-0	I-1; I-1; II, I, 4	0-3; 1, 2, 3
Leg 2	0-1	0-0	I-1; I-1; III, I, 5	0-3; 2, 2, 4
Leg 3	0-1	0-0	I-1; I-1; III, 1, 5	0-3; 2, 2, 4
Leg 4	0-1	0-0	I-1; I-1; III, 1, 5	0-3; 2, 2, 3

Leg 5 (Fig. 3E) biramous, slightly asymmetrical; coxa and intercoxal sclerite fused. Basis subrectangular, with posterior seta. Endopod rounded distally, about 0.3 times as long as exopod. Exopods of both legs 1-segmented, bifurcated tip and with 2 outer spines; outer process on left slightly larger than right and with small spine-like process on proximal part.

##### Male.

**Figure 4.**
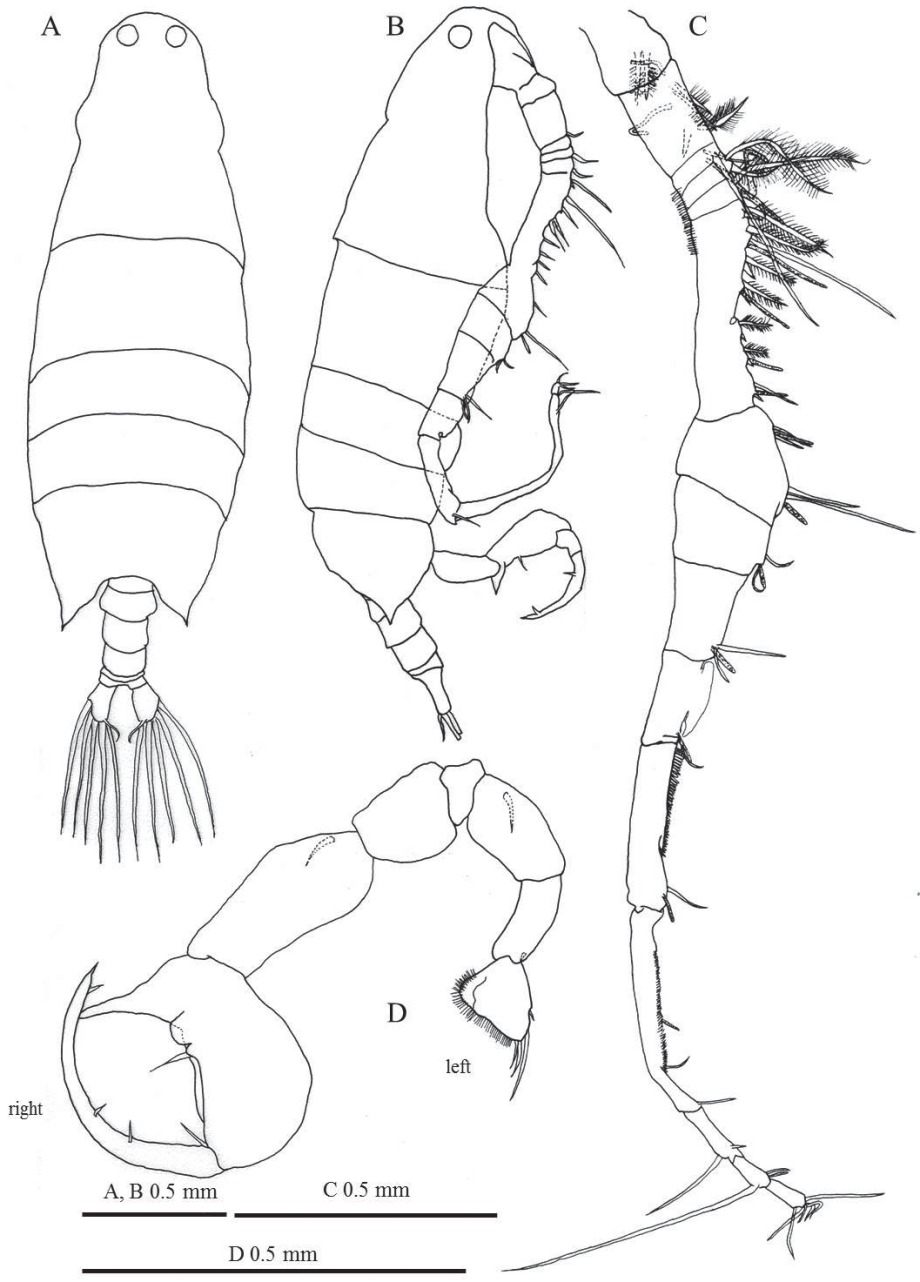
*Labidocera churaumi* sp. n., male (Paratype). **A** habitus, dorsal view **B** habitus, lateral view **C** right antennule **D** leg 5.

Body (Fig. 4A, B) slightly smaller than female (1819–2531 µm, average: 2219 µm, n=20). Prosome about 4 times as long as urosome, Urosome (Fig. 4A) symmetrical with 5 somites; anal somite and caudal rami asymmetrical.

Right antennule (Fig. 4C) with 15 segments geniculate between segments 11 and 12, reaching middle of third pedigerous somite. Antennular segments armed as follows (Arabic numbers=setae; sp=spines, aes=aesthetasc): (I) 2+aes, (II-IV) 4+aes, (V) 2+aes, (VI) 2, (VII) 2+aes, (VIII-XIV) 8+6sp+4aes, (XV-XVI) 4+2aes, (XVII) 2+aes, (XVIII) 2+aes, (XIX) 1+aes, (XX) 1+aes, (XXI-XXIII) 2+aes, (XXIV) 2, (XXV) 2+aes, (XXVI-XXVIII) 6; Segments 11 and 12 with row of teeth.

Left antennule, antenna, mouthparts and swimming legs as in female.

Leg 5 (Fig. 4D) asymmetrical. Left leg 5 short; intercoxal sclerite and left coxa fused. Basis cylindrical with seta near base. Exopod 2-segmented: first segment cylindrical; second segment triangular short with protruding hairy medial surface and 3 distal and 1 lateral spines, one of them long. Right leg 5 basis with seta. Exopod 2-segmented, forming chela; thumb of chela large, triangular, arising near base of first exopodal segment. First exopodal segment with 2 small setae. Second exopodal segment elongate and curved, with 3 slender marginal setae.

##### Remarks.

The present new species is similar to *Labidocera madurae* Scott, 1909 and *Labidocera tasmanica* Taw, 1974 in having the following features: (1) the posterolateral margins of the prosome are symmetrical, each triangular with a sharply pointed tip; (2) the female urosome is moderately or markedly asymmetrical; (3) the caudal rami are symmetrical and not highly modified; (4) the endopods of female leg 5 are nearly symmetrical, short, conical, and not bifid at the tip; (5) the thumb of the right leg 5 of the male is triangular with a broad base, and is slightly recurved; (6) the distal part of terminal segment of the left leg 5 of the male bears 3 spines, the outermost of which is the longest. These 3 species constitute a species group within the *Labidocera detruncata* species complex (see Discussion). *Labidocera churaumi* sp. n. can be distinguished from *Labidocera madurae* and *Labidocera tasmanica* by: (1) the presence of right postero-lateral and left antero-lateral processes on the female genital compound somite; (2) the exopod of the female leg 5 is very short, only as long as the basis, and has a bifurcated tip on both sides; (3) the inner margin of the terminal segment of the male left leg 5 has a protrusion at mid-length.

##### Etymology.

The new specific name “*churaumi*” is from an Okinawan dialect, meaning the beautiful seas around the type locality Okinawa.

## Discussion

Fleminger (1967) classified species of the genus *Labidocera* into four superspecies (=species complex), the *Labidocera wilsoni* Fleminger & Tan, 1966, *Labidocera detruncata* (Dana, 1849), *Labidocera darwini* Lubbock, 1853, and *Labidocera kroyeri* (Brady, 1883) species complexes, but left some species unassigned. Within the *Labidocera detruncata* species complex he recognized nine species. Subsequently Greenwood and Othman (1979) added further three species to this species complex, and compared nine Indo-West Pacific members of the species complex, but did not consider three species: *Labidocera orsinii* Giesbrecht, 1889, *Labidocera gangetica* Sewell, 1934 and *Labidocera nerii* (Krøyer, 1849) that Fleminger (1967) originally assigned to this species complex. Subsequently Greenwood and Othman (1979) and Othman (1986) added new species: *Labidocera farrani* Greenwood & Othman, 1979 and *Labidocera jaafari* Othman, 1986 to the species complex. Mulyadi (2002) was the first to define the Indo-West Pacific species group within the *Labidocera detruncata* species complex which he referred to as *Labidocera detruncata* species group, in which were accommodated the following ten species: *Labidocera detruncata*, *Labidocera pavo* Giesbrecht, 1889, *Labidocera bataviae* Scott, 1909, *Labidocera madurae* Scott, 1909, *Labidocera cervi* Krämer, 1895, *Labidocera caudata* Nicholls, 1944, *Labidocera sinilobata* Shen & Lee, 1963, *Labidocera tasmanica* Taw, 1974, *Labidocera farrani* Greenwood & Othman, 1979, and *Labidocera jaafari*. One remaining issue is that two Atlantic species (*Labidocera orsinii* and *Labidocera nerii*) and the Indian species (*Labidocera gangetica*) were not assigned to this species complex by Mulyadi (2002). Here we reinstate Fleminger’s (1967) inclusion of the two Atlantic species (*Labidocera orsinii* and *Labidocera nerii*) and the Indian species (*Labidocera gangetica*).

Therefore Mulyadi’s (2002) definition of the Indo-West Pacific *Labidocera detruncata* species group needs some emendations as follows: (1) the posterolateral prosomal corners of both sexes protrude posteriorly into a pointed tip; (2) the female urosome is slightly or distinctly asymmetrical, about 1/6 to 1/4 as long as prosome, and 2- or 3-segmented; (3) the rostrum of both sexes is widely divided; (4) the caudal rami of the female are slightly or remarkably asymmetrical, broadened, with or without one or more thickened setae; (5) the exopods of female fifth legs are asymmetrical, with each bearing 3 lateral and 2 terminal processes; (6) the endopods of female fifth legs are either simply conical (rarely bifid) distally or are totally reduced; (7) the thumb of the right leg 5 of the male is conical or spatulate; (8) the finger of the right leg 5 of the male is slender; (9) the terminal segment of the left leg 5 of the male bears 1 outer and 3 slender terminal (rarely thick in *Labidocera caudata*) elements. Since the two Atlantic and *Labidocera gangetica* comply with this emended diagnosis, Fleminger’s (1967) original *Labidocera detruncata* species complex (Mulyadi’s (2002) Indo-West Pacific *Labidocera detruncata* species group plus *Labidocera orsinii*, *Labidocera nerii* and *Labidocera gangetica*) is well defined by this amended diagnosis.

**Figure 5.**
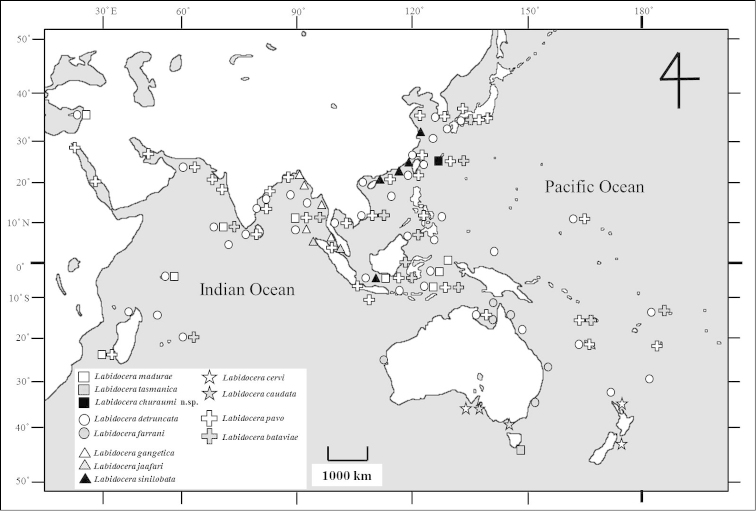
Distribution of *Labidocera detruncata* species complex based on previous records and present study.

Although Mulyadi (2002) defined the Indo-West Pacific *Labidocera detruncata* species group (the ten species listed above), it can be further subdivided into the following five newly proposed species groups on the basis of variation in the differing expressions of sexual dimorphism. *Labidocera sinilobata*, *Labidocera jaafari* and *Labidocera gangetica* share synapomorphies in variation in the differing expressions of sexual dimorphism, viz., (1) the absence of endopods from leg 5 of the female, (2) the thumb and finger of the right leg 5 of the male slender, (3) there is a rounded process present basal to the thumb of the right male leg 5, and (4) there is a protrusion on the inner surface of the terminal segment of the left male leg 5. These three species have a restricted distribution in the subtropical and tropical, coastal waters of the Indo-West Pacific (Sewell 1912, Shen and Lee 1963, Silas and Pillai 1973, Othman 1986, Mulyadi 2002, Razouls et al. 2014), and are referred to here as *Labidocera gangetica* species group (Fig. 5).

Two species, *Labidocera cervi* and *Labidocera caudata* from Oceania are unusual in both having a triangular process distally on the terminal segment of the male left leg 5. The first exopodal segment of male leg 5 bears an outer subterminal process in both species, although it is not certain whether these are homologous. In the female the exopod of leg 5 bears 3 distinct lateral and 2 terminal prominences, while the endopod is simply spiniform. The posterolateral prosomal corners of *Labidocera cervi* are remarkably large compared to those of *Labidocera caudata*. These two species are considered here as the *Labidocera cervi* species group. McKinnon and Kimmerer (1984) pointed out the similarity in the fifth legs of both sexes of *Labidocera caudata* and *Labidocera madurae*, but we regard the unique structure of the terminal exopodal segment of the male left leg 5 as a robust synapomorphy to define the species group.

*Labidocera detruncata* is most closely related to *Labidocera farrani* in sharing the following synapomorphies: (1) the complex, dorsal swelling on the genital compound somite of the female; (2) the anal somite of the female protruded mid-posteriorly; (3) the caudal rami of the female are widely separated, and the right ramus is larger than the left; (4) the basis of the female leg 5 is swollen; (5) the male right leg 5 has a spatulate thumb; (6) the terminal segment of the male left leg 5 has 4 elements, the second outermost of which is the longest. These two species belong to *Labidocera detruncata* species group *sensu stricto.* *Labidocera detruncata* is widely distributed in oceanic waters of the Indo-Pacific and West Atlantic regions, while *Labidocera farrani* has a distribution in coastal waters of Indo-West Pacific (Silas and Pillai 1973, Greenwood and Othman 1979, Mulyadi 2002, Jeong et al. 2009, Razouls et al. 2014) (Fig. 5).

*Labidocera pavo* and *Labidocera bataviae* share the following features in the female: (1) the female caudal rami are broadly separated and posterolaterally expanded; (2) the exopod of the female leg 5 is slender, with 3 lateral and 2 terminal distinct prominences; (3) the endopod of the female leg 5 is short, at most 1/3 to 1/4 as long as the exopod; (4) the thumb of the first exopodal segment of the male right leg 5 is bifid; (5) the terminal exopodal segment of the male left leg 5 is slender, and carries 3 fine elements. These two species belong to the *Labidocera pavo* species group and they are broadly distributed in coastal waters of the temperate to tropical Indo-Pacific regions (Silas and Pillai 1973, Razouls et al. 2014) (Fig. 5).

As already mentioned in “Remarks”, *Labidocera madurae*, *Labidocera tasmanica* and *Labidocera churaumi* sp. n. together belong to the *Labidocera madurae* species group. This species group has a restricted distribution in tropical to temperate waters of the Indo-Pacific (Scott 1909, Silas and Pillai 1973, Taw 1974, Razouls et al. 2014, present study) (Fig. 5). *Labidocera detruncata* is widely distributed in the Indo-West Pacific, while *Labidocera pavo* has a narrow coastal distribution in the region. In addition *Labidocera sinilobata* is restricted to the West Pacific, whereas *Labidocera gangetica* occurs in the Indian Ocean. Such restricted distributions within this species complex suggest us the possibility of parallel speciation due to the isolation mechanism by the existence of Sundaland during the glacial periods (cf. Fleminger 1986).

### Key to Indo-West Pacific species groups in *Labidocera detruncata* species complex

**Table:** 

1	Endopods of female leg 5 absent; thumb and finger of male leg 5 slender	***Labidocera gangetica* species group**
–	Endopods of female leg 5 present; thumb and finger of male leg 5 not slender	**2**
2	Female genital compound somite with complex swelling dorsally; apical segment of male left leg 5 with 4 terminal elements, second outermost the longest	***Labidocera detruncata* species group**
–	Female genital compound somite lacking dorsal swelling; apical segment of male left leg 5 with 4 or fewer elements terminally	**3**
3	Female caudal rami asymmetrical, widely separated, perpendicular to anal somite; thumb of first exopodal segment of male right leg 5 bifid	***Labidocera pavo* species group**
–	Female caudal rami symmetrical, neiwther widely separated nor perpendicular to anal somite; thumb of first exopodal segment of male right leg 5 not bifid	**4**
4	Posterolateral prosomal corners of female asymmetrical with right longer than left; terminal exopodal segment of male left leg 5 with distal triangular process	***Labidocera cervi* species group**
–	Posterolateral prosomal corners of female symmetrical; terminal exopodal segment of male leg left 5 lacking of distal triangular process	***Labidocera madurae* species group**

### Key to species of *Labidocera madurae* species group

**Female**

**Table:** 

1	Genital compound somite with right postero-lateral and left antero-lateral processes	***Labidocera churaumi* sp. n.**
–	Genital compound somite without right postero-lateral and left antero-lateral processes	**2**
2	Genital compound somite more than twice as long as wide, furnished with anterior triangular small process on each side	***Labidocera tasmanica***
–	Genital compound somite as long as wide, and produced mid-laterally	***Labidocera madurae***

**Male**

**Table:** 

1	Inner margin of terminal segment of left leg 5 with protrusion at mid-length	***Labidocera churaumi* sp. n.**
–	Inner margin of terminal segment of left leg 5 without protrusion	**2**
2	Terminal segment of right leg 5 with expanded basal region with serrated margin	***Labidocera tasmanica***
–	Terminal segment of right leg 5 without expanded basal region	***Labidocera madurae***

## Supplementary Material

XML Treatment for Labidocera churaumi
